# The Origin of Variation in Primary Care Process and Outcome Indicators: Patients, Professionals, Centers, and Health Districts

**DOI:** 10.1097/MD.0000000000001314

**Published:** 2015-08-07

**Authors:** Juan F. Orueta, Arturo García-Alvarez, Gonzalo Grandes, Roberto Nuño-Solinís

**Affiliations:** From the Centro de Salud de Astrabudua (Primary Health Care Center of Astrabudua), Osakidetza (Basque Health Service), Erandio (Bizkaia), Spain (JFO); Primary Care Research Unit-Bizkaia, Osakidetza, Bilbao, Spain (AG-A, GG); and Deusto Business School Health, University of Deusto, Bilbao, Spain (RN-S).

## Abstract

Healthcare providers are often evaluated by studying variability in their indicators. However, the usefulness of this analysis may be limited if we do not distinguish the variability attributable to health professionals and organizations from that associated with their patients.

Our objectives are to describe the main process and outcome indicators of primary healthcare services, analyzing the contribution to variability in these indicators from different levels: individual, health professional, health center, and health district.

This is a cross-sectional study that includes all.

All the individuals covered by the public Basque Health Service (children [age 0–13], n = 247,493; adults [≥14 years old], n = 1,959,682) over a 12-month period.

We calculated the number of visits to primary care doctors, number of referrals, prescription costs, and potentially avoidable hospitalizations for ambulatory care sensitive conditions (ACSCs). Using multilevel analysis, we determined the percentage of variance attributable to each level.

After adjusting for the characteristics of patients (demographic, socioeconomic, and morbidity), doctors (panel size), health center (size, staff satisfaction, demographic structure of the community), and health district, the variance in the indicators was mainly attributable to differences between patients, independently of the attending health professional, the center, or the healthcare organization, both in children (94.21% for visits to the doctor; 96.66% for referrals; 98.57% for prescription costs; 90.02% for potentially avoidable hospitalizations for ACSCs) and in adults (88.10%; 96.26%; 97.92%; and 93.77%, respectively).

The limited contribution of health professionals and organizations to variability in indicators should be taken into account when performing evaluations and planning quality improvement strategies.

## Abstract 2

Supplemental Digital Content is available in the text

## INTRODUCTION

Variation in medical practice is a topic of interest in primary as well as other levels of care. Unexplained differences in healthcare may influence equity, outcomes of care provided and efficiency in the use of resources. As a consequence, numerous initiatives have been developed to measure and analyze such differences and to understand their causes.

Both in industrialized^1^ and developing countries,^2^ it is common to compare indicators obtained in populations under the care of primary care professionals. The results of this profiling have been used as a training and motivational tool for doctors (telling them their position with respect to their colleagues), to give financial rewards to those achieving good outcomes, and to guide patients toward the best-performing providers.^3^

In this context, since 2005, in the Basque Country (Spain) the Basque Health Service (Osakidetza) has used an annual evaluation system that assesses primary care doctors, health centers, and healthcare districts in terms of efficiency in the use of healthcare resources and quality of care. In order to compare apples to apples, this assessment is adjusted for age, sex, and morbidity in the populations seen, using the Adjusted Clinical Group (ACG) Case-Mix System developed at Johns Hopkins University.^4^

The logic behind this is that, once we have adjusted for differences between patients, variations in outcomes are attributable to medical practice, and hence, said outcomes may be modified directly, by acting on the way health professionals work. However, the usefulness of this approach may be limited, unless we take into account the hierarchical nature of data.^5^ Patients are not isolated units, independent of one another; rather they are individuals grouped in doctors’ lists, and in turn, doctors are based in particular health centers and healthcare organizations. Although some studies have indicated the need to use hierarchical statistical models for making comparisons between hospitals,^6^ health centers,^7^ and doctors,^3,7,8^ very few publications to date have analyzed the contribution of different levels of care to variability in primary care indicators.

In this study, we used the data from all Osakidetza primary care physicians providing healthcare to more than 2 million people, almost the entire population of the geographical area under study (Basque Country, Spain). Our objective was to describe the main indicators for measuring resource use (visits to doctors, referrals, and prescriptions), healthcare outcomes (potentially avoidable hospitalizations) and their relationship with factors related to patients, doctors, and health centers, as well as to ascertain the contribution of each of these levels to the variability observed in these indicators.

## METHODS

This was a cross-sectional study, analyzing healthcare process and outcome indicators in the public health service in the Basque Country over a period of 1 year. The protocol was approved by the Clinical Research Ethics Committee of the Basque Country (reference number: 11/2012).

### Setting

Osakidetza is a public organization funded by taxes. It provides near-universal coverage for the population living in the Basque Country. Care is free at the point of delivery except for prescriptions, for which there is copayment, this varying with the type of disease and the patient's social and employment status.

At the time of this study, in 2008, primary care health services were organized into 7 health districts, corresponding to geographical areas. Each health district was composed of 9 to 22 health centers. Funded by annual contracts with the Department of Health of the Government of the Basque Country, health districts are organizationally independent and accountable in economic terms.

Primary care physicians are public sector workers, receiving a salary and a capitation payment, and work in multidisciplinary care teams. Every inhabitant is included on the list of a doctor, who is a pediatrician or a family doctor depending on the patient's age (<14 vs ≥14 years old). These primary care doctors act as gatekeepers to other levels of care. Electronic health records (EHRs) started to be introduced in 1990 and are now used by all primary care doctors.

### Study Population and Period

The observation period was set at 1 year, from September 1, 2007 to August 31, 2008. In such period, the total population covered by the Basque public healthcare system was 2,265,058 individuals. We considered that the primary care doctor was responsible of the care of a patient if such person was registered in his/her list most part of the year of study (ie, 185 or more days), independently whether they had any type of contact. Due to several reasons (migration, dying, newborn, and multiple changes of physician or administrative modifications) 57,883 people (2.6%) did not complete such 6-month period with the same doctor and were excluded from our analyses. So, the study population included almost the entire population of the Basque Country.

In this study, we analyzed the data from across the public primary care network: 130 health centers, 1193 family doctors, and 286 pediatricians. The total number of registered inhabitants was 2,207,175 of whom 247,493 (11.2%) were children (age<14 years).

The extent of the relationship between providers and patients can influence the quality of care and its outcomes. Inclusion of individuals with very short enrollment periods can affect the results of some providers and, therefore, the Basque Health Service has established 185 days as the minimum period to consider that a provider (physician, health center, or health district) is accountable for the care provided to a patient in their annual evaluations. Accordingly, we kept such criteria in our study. However, there is not a unanimous opinion between researchers about the decision of include/exclude patients with partial enrollment. So that, we repeated all our analyses limiting the study population to the 1,901,927 people that remained the full 12-month period with the same physician. Such results were very similar to the ones presented in this article and are available on demand for interested readers (please contact the authors for further information).

### Sources of Data

We use the following sources of data:

- Primary care EHRs of the Basque public health system, containing demographic, administrative, and clinical data including diagnoses, prescriptions, ancillary examinations, and referrals generated in each patient visit.
- The minimum basic data set, gathering information regarding all the patient discharges from all hospitals across the Basque public health network, including data on patient characteristics, hospitalization episodes, diagnoses, and procedures.

### Variables

The explanatory variables used at the patient level were demographic characteristics (age and sex), morbidity (primary and secondary diagnoses), and socioeconomic status (census-based deprivation index).

In order to include a manageable number of disease categories, all the patient diagnoses (ICD-9-CM codes used by the primary care doctors during the year of study) were classified into Aggregated Diagnosis Groups (ADGs). The ADG system assigns ICD-9-CM codes to 1 of 32 categories, as a function of clinical criteria, expected resource use and the type of care likely to be required for each health problem. It is part of the Johns Hopkins population-based ACG Case-Mix adjustment system.^4^ In our study, we applied a “lenient” diagnostic certainty option: any single diagnosis included in an ADG was enough to accept the corresponding ADG for such patient.

The census-based deprivation index, used as a proxy for the socioeconomic status, was developed for the MEDEA project (Mortalidad en áreas pequeñas Españolas y Desigualdades socio-Económicas y Ambientales—Mortality in small Spanish areas and socio-economic and environmental inequalities).^9^ In Spain, census tracts are the smallest geographical units into which census data are divided; they are mainly defined by criteria related to population size but also reflect natural and man-made features. Although the number of people living in a tract is variable, the median population per census tract is around 1200 people.

This index is constructed from variables related to employment (rates of unemployment, and of manual and short-term employment) and education (low rates of educational attainment among young people and overall). In this study, deprivation index scores were categorized into quintiles (the 5th corresponding to areas with the greatest deprivation, and the 1st to the least deprived areas). It is a measure of the ecological effect on individuals of dwelling in a census tract and provides an indicator of the level of access to economic and material resources in a community. It has been shown to be correlated with mortality rates^10^ and prevalence of morbidity.^11,12^

At the doctor level, we considered the number of patients on a doctor's list to estimate his/her workload.

To analyze the characteristics of the primary care centers we included variables relative to demographic characteristics of the area (percentage of the population above 65 years of age and percentage of immigrants in the corresponding geographical areas),^13^ size of the center (number of primary care doctors), and satisfaction of the staff with their working environment. For the last of these variables, we used the overall satisfaction scores for health centers (on a scale of 0–10), taken from internal surveys that Osakidetza regularly carries out in all its organizations.^14^ As for the doctors, the health centers were categorized into quintiles. Categorization of these variables was necessary because some of the statistical models did not achieved convergence when we used them as continuous.

The outcome variables were the number of visits to primary care doctors, number of forms for referrals to specialized care issued to users by these doctors, and cost of medications prescribed by primary care physicians and paid for by the Department of Health during the year of study. In addition, we identified patients with hospitalizations for ambulatory care sensitive conditions (ACSCs) that might have been avoided had they received appropriate and timely outpatient care. For ACSCs, we used the list developed by Casanova Matutano et al^15^ for the pediatric population and that of Caminal et al^16^ for the rest of the population.

### Statistical Analysis

The study population was divided into 2 groups that were analyzed separately: individuals on pediatricians’ lists and those on family doctors’ lists. The methodology for the analysis was identical for the 2 groups. All analyses were carried out using SAS (SAS/STAT, version 9.2; SAS Institute, Inc., Cary, NC, USA).

Four-level mixed effect models^5^ were used to assess the relationship between the various different outcome variables and the characteristics of patients, doctors, and health centers. Taking into account the hierarchical nature of the data, these models include each of the explanatory variables as fixed effects and random intercepts for each of the levels (the patient, doctor, health center, and health district).

For prescription costs, we built generalized linear mixed models (GLMMs) with gamma distribution and log link.^17^ As healthcare cost data are typically nonnormally distributed with a skew toward the right, Gamma regression is a better modeling approach to deal with this skewness than ordinary least square.^18–20^ In the case of visits to the doctor and referrals, although Poisson regression is often employed for this type of outcomes, we used GLMMs with negative binomial distribution as our data showed signs of over-dispersion.^21^ Using likelihood ratio tests we checked that the negative binomial regression models provided a better fit than did the Poisson models.^21^ Finally, the existence of an ACSC hospitalization is a binary variable (yes/no), and therefore was modeled using logistics GLMMs.^22^ We rescaled the fixed and random effects using the method developed by Hox,^23^ because in multilevel logistic models the variance of the residual variance (at the individual level) is fixed at a constant.

All the models were developed using PROC GLIMMIX with the Laplace method. Effect sizes of associations were exponentiated and reported as ratios. We consider an association as significant when the *P* value was below 0.05.

For each outcome variable we calculated the intraclass correlation coefficients (ICCs) and percentages of variance at each level, both for empty models (no explanatory variables, random effects analysis of variance [ANOVA]) and for adjusted models (full set of explanatory variables).

In the case of prescriptions cost, visits to the doctor and referrals we used the method of normal approximation treating the outcomes as if they were normally distributed variables and analyzing them employing linear mixed models (PROC MIXED).^24^ Using the variance components obtained with these models, the calculation of the percentages of variance and ICCs at each level is straightforward. In a multilevel model, the ICCs are estimated as the ratio between the variance at the level (which include the variance at its higher levels) and the total variance. The ICCs can be interpreted as the correlation among observations within the same cluster (eg, correlation of 2 patients in the same health center).^23^

For ACSC we used logistic mixed models, which provide the variance components for the nonindividual levels (doctor, health center, and health district), and the formula of Snijders,^22^ based on a latent continuous variable that considers that the variance attributable to individuals is approximately 3.29.

## RESULTS

### 

Patient, physician, and health center level characteristics are presented for adult and pediatric patients in Table 1. Table 2 shows the unadjusted outcomes: visits to the physician, referrals, cost of prescriptions, and ACSC.

By applying multilevel analysis, the demographic characteristics of patients had an influence on resource use and hospitalization for ACSCs, although not homogeneously. In the adult population (Table 3 ), the number of visits to the doctor and prescription costs increased with age, until 84 years of age, at which point they decreased slightly; while in the case of referrals to a specialist, such reduction was observed at 65 years of age and beyond; we found a bimodal distribution in ACSC hospitalizations with a peak among younger patients and another in the most elderly ones. Among the children (Table 4), the rates of visits to primary care pediatricians and of hospitalization for ACSCs decreased with age, while the opposite trend was observed for referral rates and prescription costs. With respect to sex, both in adults and children, prescription costs per patient were higher among male patients, and they visit their primary care doctor less often than females. Nevertheless, adult men were less often referred to specialists and were more likely to be hospitalized for ACSCs than women; in children, there were no significant differences in referrals and the opposite pattern was found in hospitalizations.

Although the differences were small, the least disadvantaged socioeconomic groups presented less frequent visits to the primary care doctor and referrals; for prescriptions, there were not differences in adults and the rates of hospitalizations for ACSCs were smaller among individuals living in richer areas; in pediatric patients, although being statistically significant their differences, there was no clear gradient for prescriptions and ACSC hospitalizations.

In adults, diagnoses in any of the 32 diagnostic groups (ADGs) were associated with more visits to the primary care doctor and referrals (see Supplementary Materials, http://links.lww.com/MD/A371). This pattern was also observed for children, except 1 group in visits and 2 in referrals for which the differences were not statistically significant. Regarding prescriptions, we found a higher use of drugs in 30 of the ADGs in both populations. The risk of potentially avoidable hospitalization in adults was higher for 20 ADGs and lower for 5 ADGs, while in children, the risk was higher for 14 ADGs and lower for 3.

Concerning variables related to the doctor (panel size) and center (size, staff satisfaction, percentages of immigrants, and elderly people in the population), we did not find any statistically significant trends (Tables 3 and 4), with 2 exceptions in adults: patients in small panels receive less prescriptions and the percentage of aged population that achieve statistical signification for referrals but does not show a defined gradient.

In the empty models, the variance observed was almost exclusively due to differences between patients (children or adults). Specifically, the percentage of variance associated with health professionals, and hence, health centers and districts, was <3% for prescriptions, 5% for referrals, and 9% for visits to the primary care doctor, being around 7.4% and 3.8% for potentially avoidable hospitalizations in children and adults, respectively (Tables 5 and 6). After adjusting for all the explanatory variables, there was a significant decrease in total variance: in adults, by more than 50% for visits to the primary care doctor and by around 30% for the other dependent variables; and in children, by 58% for visits, 23% for potentially avoidable hospitalizations, 16% for referrals, and 14% for prescriptions. However, we did not find relevant changes in the percentage of the unexplained variances attributable to levels higher the patient, and, in fact, their sum only reaches figures of over 5% for visits to the primary care doctor (12.2% in adults; 6.2% in children) and for potentially avoidable hospitalizations (children 10.3%; adults: 6.1%).

### 

**TABLE 1:**
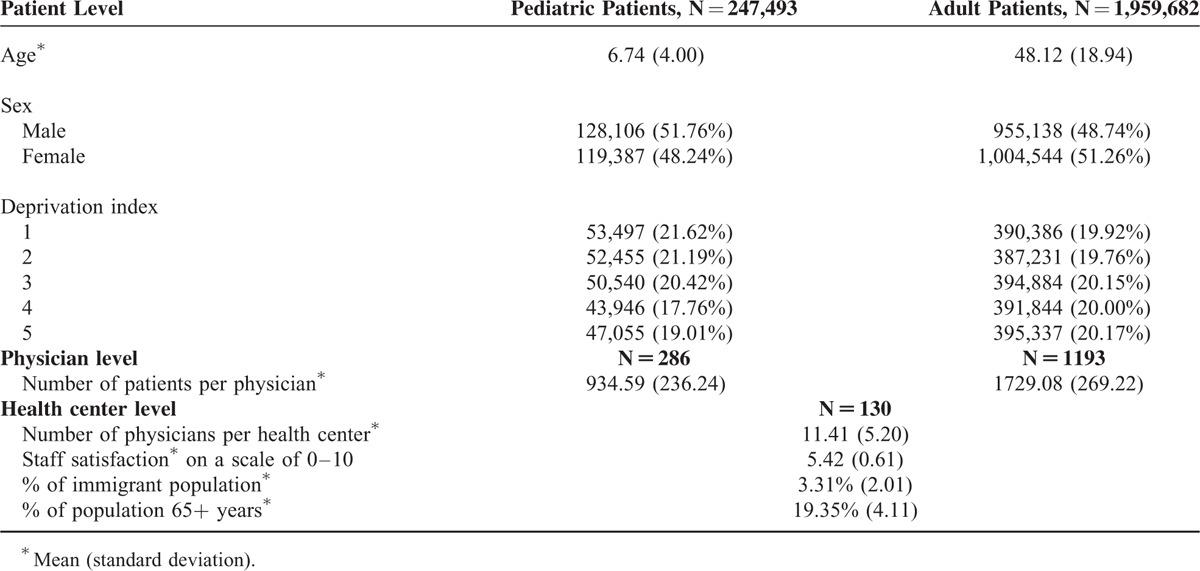
Patient, Physician, and Health Center Level Characteristics

**TABLE 2:**
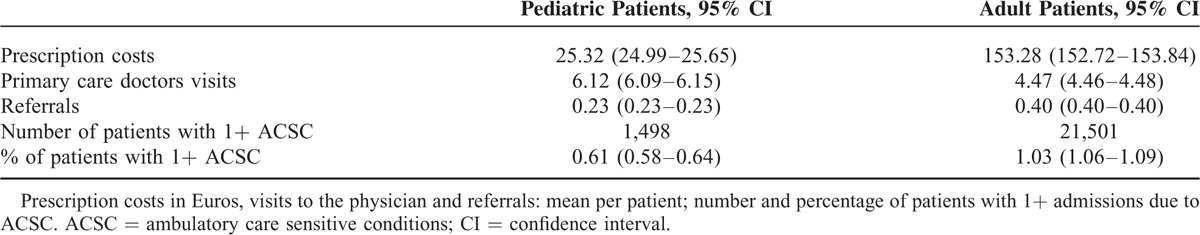
Unadjusted Outcomes

**TABLE 3:**
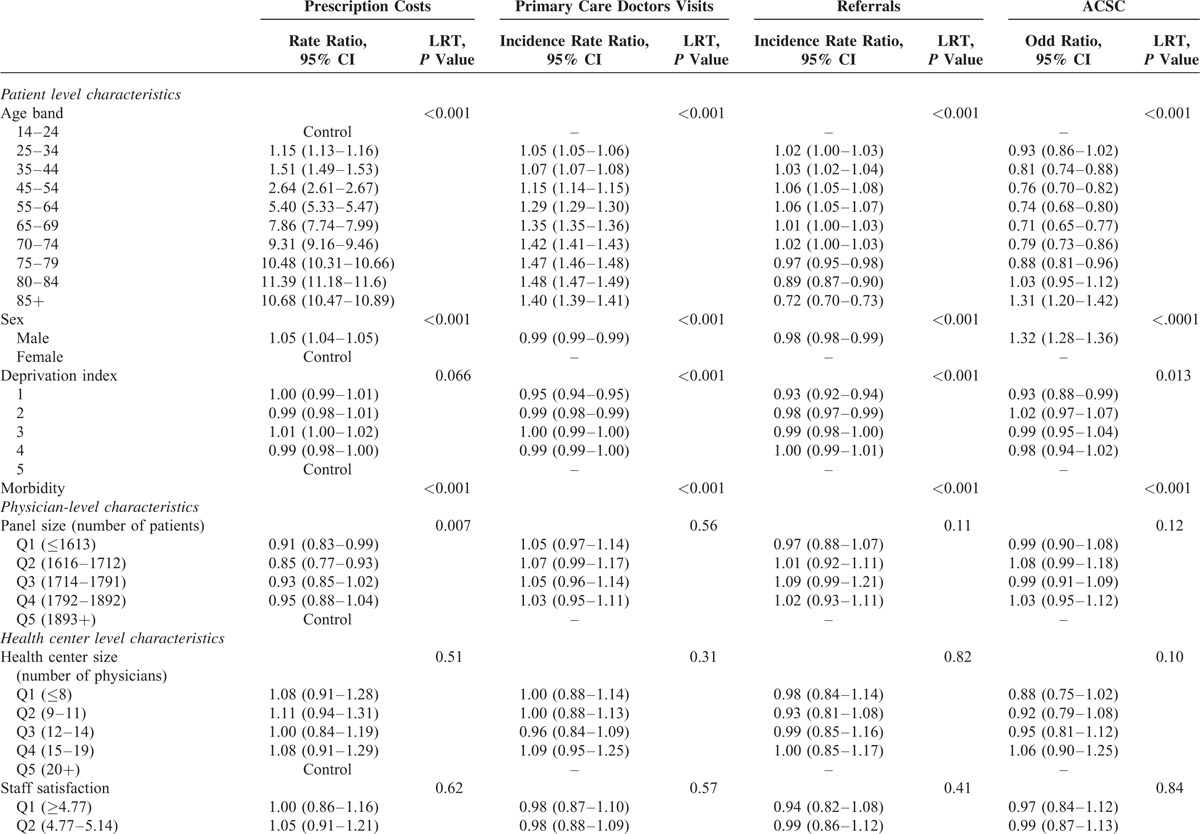
Multilevel Analysis: Fixed Effects Adult Patients (Age 14+)

**TABLE 3 (Continued):**
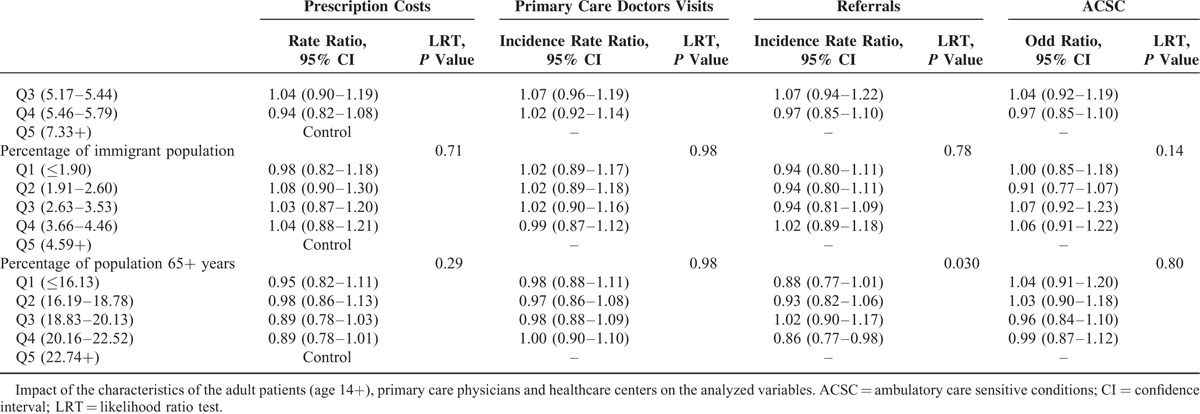
Multilevel Analysis: Fixed Effects Adult Patients (Age 14+)

**TABLE 4:**
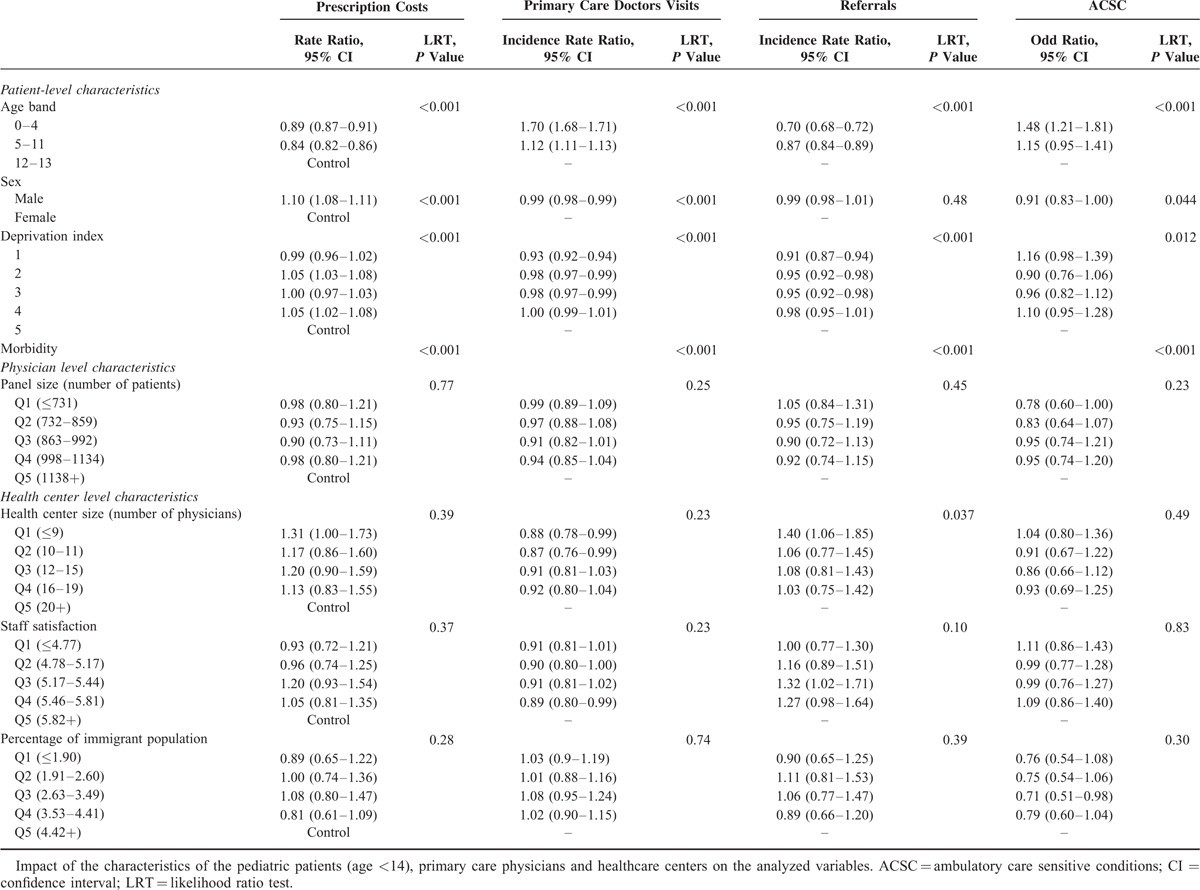
Multilevel Analysis: Fixed Effects Pediatric Patients (Age <14)

**TABLE 5:**
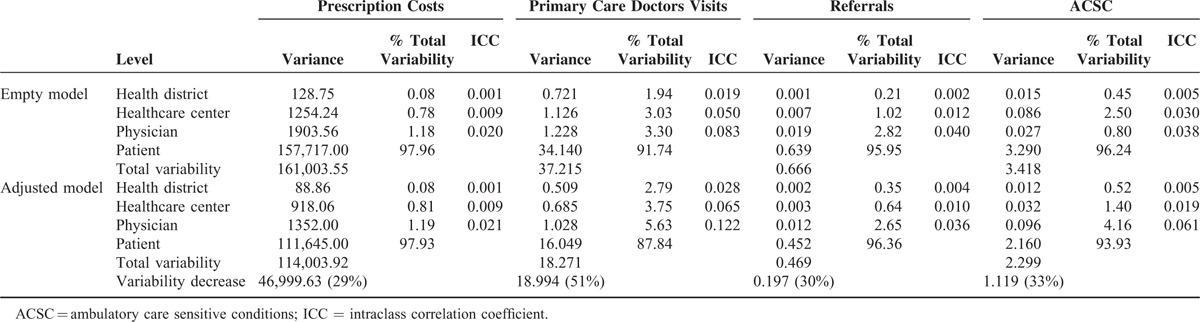
Proportion of Variability Attributed to Patients, Physicians, Primary Healthcare Centers, and Districts: Adult Patients (Age 14+)

**TABLE 6:**
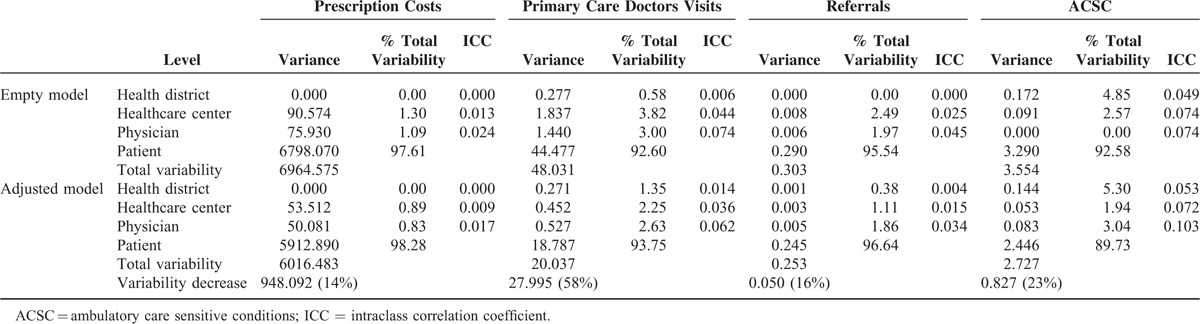
Proportion of Variability Attributed to Patients, Physicians, Primary Healthcare Centers, and Districts: Pediatric Patients (Age <14)

## DISCUSSION

Our results demonstrate that only a small proportion of variability in costs and in healthcare outcomes can be attributed to doctors or health centers. Even using a sophisticated and widely validated population-adjustment system, a significant percentage of the overall variance remains to be explained; it is attributable to patients but cannot be explained by their morbidity or demographic characteristics.

Other authors have also observed that patient characteristics determine most of the variability observed both between doctors and between levels of care. Hofer et al^7^ estimated that the effect attributable to doctors’ way of working accounted for only 4% of the variance in visits of patients with diabetes mellitus, 1% in hospitalizations and 3.3% in glycemic control. Comparing hospital indicators, other authors found that the percentage of the variance attributable to health centers did not even reach 1% in the rates of readmission within 30 days^6^ and was around 10% in terms of use of intensive care resources.^25^ In a systematic review, Fung et al^8^ analyzed 21 studies that provided estimates of the percentage of variance explained by different levels of care, using hierarchical models; although results were mixed (in part attributable to methodological differences), the total variability attributable to the levels higher than the patient (doctors’ list, center, hospital, or healthcare plan) was low, in almost all cases, when analyzing process or outcome measures, and higher considering patient satisfaction. More recently, it has been reported that 85% of the variance in outcomes in terms of overall technical quality in primary care centers^26^ and 92% of differences in the adequate prescription of beta blockers after a heart attack^27^ are attributable to patient-level factors.

In our analysis, we did not find any associations of family doctor's panel size or work satisfaction, or health center size with outcomes in terms of healthcare resource use or ACSC hospitalizations in pediatric patients; in adults, we only observed more prescriptions in doctors with large panels, as well as differences in referrals to the specialists among health centers according to the percentage of elderly people in the geographic area, but without a defined gradient. Although some researchers have found an association between the satisfaction of primary care doctors and the provision of better quality care^28^; this has not been confirmed in other studies.^29,30^ Further, there is no consensus on what is the most suitable size for a primary care health center to achieve the best healthcare outcomes.^26,31^

In healthcare organizations, it is important to record indicators of healthcare providers and analyze their variability, as they can help monitor the quality of the healthcare provided. In particular, it is unquestionable that, in this way, we may be able to detect inefficiencies in medical practice, clinical errors, and healthcare gaps, but this task is not straightforward and often variability can be attributed to the adaptation of the healthcare provided to the individual needs of patients or to the participation of patients in decision making.^32^ Wennberg classified clinical care into 3 categories: effective, patient preference sensitive, and supply sensitive, largely due to the capacity of the local health services.^33^ He defined unwarranted variation as care that is not consistent with patient preferences or related to patient's underlying diseases.^34^ However, as other authors recognize, discriminating between warranted and unwarranted variation can be challenging and interventions designed to reduce the observed variability risk eliminating variations that exist for good reason.^35^

In relation to this, primary care practice reports providing data on the results individual doctors has been criticized, because, among other issues, they only take into account very specific aspects of healthcare and do not provide a real measure of the complexity of the work of a primary care doctor.^36^ On the other hand, publishing such reports does not, in itself, ensure improvements in quality or motivate doctors to put in place measures to solve problems related to quality; in fact, an excessive emphasis by managers on reducing variability may encourage physician to “de-select” complex patients, declining to see the most sick patients, those whose treatments have failed or who do not adhere to treatment plans.^7^ Consequently, we believe that our findings help to put these indicators in context, since we show that only a small proportion of the variability can be attributed to healthcare professionals or the organization of health centers.

However, a small proportion does not equate to no effect at all. If we translate the differences observed into costs related to prescriptions or visits to the doctor, we find significant absolute values that justify managerial interventions. Moreover, even recognizing that the greater part of the variability is attributable to patient-level factors, it may be easier and more efficient for healthcare organizations to design actions focused on their staff rather than on patients. Further, it has been suggested that doctors may be able to influence some important factors at the patient level.^8^

The present study has some limitations. First, the data analyzed are taken from the healthcare information systems of the Basque Country, and may contain some incomplete or inaccurate information, as is always the case when using data from administrative databases or notes in EHRs. On the other hand, factors other than those analyzed may contribute to patient variability, such as those related to the family environment, psychosocial factors, or health-related habits,^37^ the same being true of the other levels studied. Besides, the socioeconomic variable used (deprivation index) may reduce the individual contribution to socioeconomic characteristics, given its ecological nature. Finally, the variability in other process or outcome indicators may be different to that of those analyzed in this study.

To conclude, our study found that doctors and health centers make only a modest contribution to variability in resource use and healthcare outcomes. This fact should be taken into account when evaluating healthcare professionals, trying to modify their behavior, and attempting to improve the efficiency and quality of organizations. Compared to traditional analysis, complex statistical methods are more costly, require more time, and are more difficult for clinicians and managers to understand. Nevertheless, a greater understanding of the factors involved in variability and a greater reliability in the estimates will help the evaluations of healthcare providers to become accepted. Given all this, strategies for improving clinical practice should include multilevel interventions that go beyond the health professionals and include individuals themselves and the organization of healthcare services.^38^
